# Notes and News

**Published:** 1896-05-30

**Authors:** 


### Notes and News.

(Collected and Communicated.)

At the Festival Dinner of the City of London Truss Society donations amounting to £735 were announced.

The late Mr. Thewlis Johnson, of Manchester, has bequeathed £500 each to the Manchester Royal Infirmary, the Ancoats Hospital, and the Derbyshire Royal Infirmary.

Through the auspices of the Epidemiological Society of London it is proposed to establish a Jenner Memorial medal for the encouragement of epidemiological research.

At the British Medical Association, to be held this year at Carlisle, the following subjects will be discussed and papers read upon them:—"The Better Governance of a Provincial Infirmary"; "The Abuse of the Out-Patients' Department in Hospitals"; "Provident Dispensaries"; "The Local Organisation of the Profession"; "Chemists and Counter Prescribing"; "The General Medical Council"; "The Overcrowding of the Profession"; "The Ethics of Midwifery Practice in Relation to Midwives"; "The Relations of Family Practitioners to Consultants"; "Medical Aid Associations."

The new departure which H.R.H. the Prince of Wales has consented to make in connection with the Guy's Hospital dinner, is likely to make that festival produce a larger sum than is usually obtained on such occasions, even under such Royal Presidency. The reception to be held by the Prince of Wales at the Imperial Institute after the dinner on the 10th prox. will be the event of the season, and the demand for tickets, the price of which has been fixed at three guineas for two tickets, is steadily increasing, and the number of distinguished people present seems likely to be so great as to make it a memorable event in the history of charity in this country. The number of tickets is necessarily limited. Few better opportunities of seeing the leading personages of the day under the most favourable conditions have ever been afforded, and foreigners, citizens of the American States, and country cousins who are able to appreciate a good thing, should apply for tickets to the secretary of Guy's Hospital forthwith.

Mr. W. J. Soulsby, whose name appears amongst the C.B.'s in the Birthday List of Honours, has acted as private secretary to successive Lord Mayors for twenty-one years. He was educated at the City of London School and King's College, and was called to the Bar at the Middle Temple in 1874. Mr. Soulsby is also a Knight Commander of the Saviour of Greece and of the St. Sava of Servia, and a Knight of the Legion of Honour of France, of Francis Joseph of Austria, of Leopold of Belgium, of the Immaculate Conception of Portugal, of the Rising Sun of Japan, and of the Takovo of Servia. He also received a Jubilee medal on the occasion of Her Majesty's visit to the Mansion House in 1877. The hospitals have no better friend in the country than Mr. Soulsby, who is ever ready to help them to the utmost, and whose efforts have often been fruitful in results. The high distinction conferred upon Mr. Soulsby by Her Majesty the Queen is deservedly the most popular of all the honours the list contains.

The Rev. C. Parkhurst Baxter, M.A., has been appointed the organising clerical secretary of the British and Foreign Sailors' Society.

Mr. Colman, of Norwich, has presented the governors of the Jenny Lind Infirmary with a site for a new infirmary.

Amongst the Birthday list of honours are to be found the names of Brigade-Surgeon A. S. Lethbridge, Brigade-Surgeon Benjamin Franklin, Dr. J. Rogers Pasha, and Surgeon-Major-General C. D. Madden.

The thirteenth annual meeting of the Paddington Green Children's Hospital will be held at the hospital, Paddington Green, on Tuesday, June 9th, at five o'clock.

The honorary degree of Doctor of Medicine has been conferred upon Sir Joseph Lister by the Emperor of Austria on the celebration of the Hungarian millennium.

The new journalism has something to answer for, and there is cause to feel that the halfpenny evening newspaper may soon have even more. We are informed by a hospital surgeon that the fathers of the poorest families in London, from whom hospital patients are largely drawn, insist on their wives supplying them with a dinner each day in accordance with the menu now published in the halfpenny newspapers. The consequence is that, when the patients are admitted to the hospital, they are greatly dissatisfied because no such menu is in common use there, and they consider the hospital diet unsatisfactory in consequence. One patient, who had been sent to a convalescent home, complained that the food was very coarse. On being asked the reason, she explained that the potatoes were served up in their jackets! Some hospitals have cooking schools attached, for the training of nurses in the culinary art; and it is to be expected that, before long, committees and medical boards will have to make themselves acquainted with the halfpenny menu, or the newspapers may be flooded with complaints by hospital patients who object to be fed except on the menu principle.

The epileptic colony at Chalfont St. Peter, near Rickmansworth, is extending rapidly, thanks to the generosity of Mr. Passmore Edwards, who is acting towards it as a veritable fairy godfather. The foundation-stone of a new home, intended for women, was laid on Tuesday by Lord Addington with "Masonic honours," and, besides contributing £2,500 towards its erection, Mr. Edwards has promised another home for children very shortly. There were many visitors at Tuesday's ceremonial. Masonic "brethren" were there in force, duly provided with the collars and aprons of their Order, and spectators might have been pardoned a wish that their garb could be completed by something more appropriate than modern dress and tall silk hats. Mr. and Mrs. Passmore Edwards were present, and among the guests were also Archdeacon Sinclair, Mr. Montefiore Nicholls (chairman of the Executive Committee), Sir Samuel Lewis, General Moberly, Dr. and Mrs. Buzzard, Miss Burdon Sanderson, Colonel Montefiore, Dr. Shuttleworth, and Dr. and Mrs. Ferrier. There are now thirty-six colonists, by whom all the work of the farm is done. Carpentering and bootmaking are carried on, too, and the joinery of the new building will be done on the spot by the inmates. It is hoped to establish a jam factory and dairy before long, and a laundry for the women to work in. For the latter and for the establishment of a resident medical officer funds are urgently needed.

